# Oxygen Plasma Modified Carbon Cloth with C=O Zincophilic Sites as a Stable Host for Zinc Metal Anodes

**DOI:** 10.3389/fchem.2022.899810

**Published:** 2022-04-28

**Authors:** Baozheng Jiang, Wenbao Liu, Zhilong Ren, Rongsheng Guo, Yongfeng Huang, Chengjun Xu, Feiyu Kang

**Affiliations:** ^1^ Shenzhen Geim Graphene Center, Tsinghua-Berkeley Shenzhen Institute and Tsinghua Shenzhen International Graduate School, Tsinghua University, Shenzhen, China; ^2^ School of Environmental and Material Engineering, Yantai University, Yantai, China; ^3^ State Key Laboratory of New Ceramics and Fine Processing, School of Materials Science and Engineering, Tsinghua University, Beijing, China

**Keywords:** zinc-ion battery, zinc anode, oxygen plasma, carbon cloth, zincophilic sites

## Abstract

Aqueous zinc-ion batteries (ZIBs) are currently receiving widespread attention due to their merits of environmental-friendly properties, high safety, and low cost. However, the absence of stable zinc metal anodes severely restricts their potential applications. In this work, we demonstrate a simple oxygen plasma treatment method to modify the surface state of carbon cloth to construct an ideal substrate for zinc deposition to solve the dendrite growth problem of zinc anodes. The plasma treated carbon cloth (PTCC) electrode has lower nucleation overpotential and uniformly distributed C=O zincophilic nucleation sites, facilitating the uniform nucleation and subsequent homogeneous deposition of zinc. Benefiting from the superior properties of PTCC substrate, the enhanced zinc anodes demonstrate low voltage hysteresis (about 25 mV) and stable zinc plating/stripping behaviors (over 530 h lifespan) at 0.5 mA cm^−2^ with 15% depth of discharge (DOD). Besides, an extended cycling lifespan of 480 h can also be achieved at very high DOD of 60%. The potential application of the enhanced zinc anode is also confirmed in Zn|V_10_O_24_·12H_2_O full cell. The cells with Zn@PTCC electrode demonstrate remarkable rate capability and excellent cycling stability (95.0% capacity retention after 500 cycles).

## Graphical Abstract

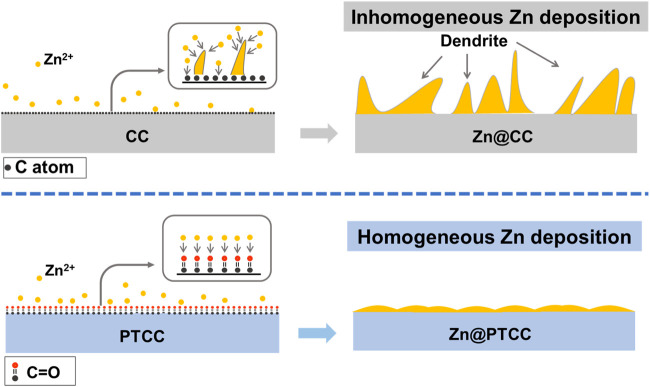

## Highlight

• Dendrite-free and stable Zn plating/stripping was achieved for over 530 h with low voltage hysteresis (25 mV) at a high DOD (15%).
• The C=O functional group introduced by oxygen plasma treatment can act as uniformly distributed nucleation sites to induce uniform zinc nucleation and deposition.
• The facile and universal plasma treatment method can also be applied to other carbon-based or organic substrates of rechargeable metal anodes.

## 1 Introduction

Energy storage devices are playing quite an essential role in human society. Lithium-ion batteries (LIBs) have achieved substantial commercial success in the last decades due to their high energy density and long cycling life (Yoshino, 2012; Liu et al., 2018; Liu Q. et al., 2020; Zu et al., 2021). However, the high cost, limited lithium resources, and safety issues have prevented LIBs from further applications (e.g., gird energy storage system) (Liu B. et al., 2020; Han et al., 2021). Zinc-ion batteries (ZIBs) are considered as one of the prospective alternatives to LIBs due to their safety and low cost. Zinc metal anode has a high theoretical specific capacity (820 mAh g^−1^) and low redox potential (−0.762 V vs. SHE) (Xu et al., 2012; Kundu et al., 2016). Besides, zinc metal also has the merits of low toxicity, high abundance, and environmental benignity. However, the development of ZIBs is still hindered by lacking stable zinc metal anodes. At present, zinc metal anodes still suffer from inherent problems of dendrite growth, zinc corrosion, and side reactions (hydrogen evolution, etc.) (Yang et al., 2019; Zhang et al., 2020a; Cai et al., 2020). The uncontrolled growth of zinc dendrites may penetrate the battery separator, leading to dead zinc and battery short circuit, which seriously hinders the commercialization of aqueous Zn-based batteries (Zeng et al., 2019; Gao et al., 2021; Mo et al., 2021). Many strategies have been developed to inhibit the growth of zinc dendrites, including electrode design, construction of artificial interface layer, electrolyte engineering, and regulation of zinc-ion flux (Kang et al., 2019; Zhao et al., 2019; Guo et al., 2020; Ma et al., 2020; Cao et al., 2021a; Zeng et al., 2021). The growth of zinc dendrites usually derives from inhomogeneous distribution of zinc ions and heterogeneous nucleation of zinc on the anode surface (Hou et al., 2021; Liu et al., 2021). To induce even nucleation and uniform deposition of zinc on the substrate, regulating the distribution and zinc bonding ability of nucleation sites is an effective strategy (Cao et al., 2021b; Xie et al., 2021).

Carbon materials are widely used in energy storage applications due to its advantages of lightweight, high conductivity, high specific surface area, and excellent flexibility (Zhou et al., 2018; Pathak et al., 2021). However, carbon materials are generally hydrophobic and zincophobic (poor ability to bond zinc), which limits their application as host material for zinc plating/stripping (Zhang et al., 2020b; Xie et al., 2021). Wang and co-workers demonstrated that a trace amount of Zn^0^ in the carbon substrate framework could provide uniform nuclei for zinc deposition and improve the reversibility of zinc plating/stripping (Wang et al., 2019). Xue et al. proposed that inkjet-printed silver nanoparticles on three-dimensional carbon framework can act as heterometallic seeds for zinc deposition and guide the uniform zinc nucleation (Chen et al., 2021). Qiao et al. revealed that the bonding between zinc ions and zincophilic sites is the mechanism for zincophilic nucleation in zinc metal anode host (Xie et al., 2021). However, these methods of introducing zincophilic sites reported in literature are relatively complicated, and the amount and distribution of the zincophilic sites are difficult to control precisely.

Therefore, we propose a simple and versatile strategy to modify the interfacial chemical state of carbon cloth (CC) through oxygen plasma treatment. The plasma treated carbon cloth (PTCC) was utilized as a host for zinc deposition. The uniformly distributed C=O functional groups were introduced to the surface of the carbon cloth by oxygen plasma treatment, which improved the electrolyte wettability and zincophilicity of the substrate, and reduced the zinc nucleation overpotential. Meanwhile, the uniformly distributed C=O functional groups can also act as active sites for zinc nucleation, guiding the uniform nucleation and deposition of zinc. As expected, the PTCC electrode exhibited a high average Coulombic efficiency (CE) of 97.42%. The Zn@PTCC anodes demonstrated low voltage hysteresis (25 mV) and stable zinc plating/stripping behaviors (over 530 h lifespan) at a current density of 0.5 mA cm^−2^ with ∼15% depth of discharge (DOD). Even at a very high DOD of 60%, the symmetric cell can still cycle steadily for over 480 h. When paired with V_10_O_24_·12H_2_O cathode, the full cell with Zn@PTCC anode exhibited superior cycling stability (95.0% capacity retention after 500 cycles), which was much higher than that of Zn@CC anode (80.1%).

## 2 Experimental

### 2.1 Materials

#### 2.1.1 Carbon Cloth and Plasma Treated Carbon Cloth

Carbon cloth (CC) was purchased from CeTech Co. Ltd. The CC was washed with deionized water and ethanol before treatment. To obtain oxygen plasma treated carbon cloth (PTCC), a plasma cleaner (PT-5ST, Sanhoptt) was used. Oxygen plasma was introduced with an RF generator, operating at a frequency of 13.56 MHz and a power of about 300 W. Pristine carbon cloth was treated with oxygen plasma for 300 s to obtain PTCC sample.

#### 2.1.2 Fabrication of Zn@CC and Zn@PTCC Electrode

The Zn@CC and Zn@PTCC electrodes were fabricated by using the constant current electrodeposition method. The CC (or PTCC) sample was used as the working electrode during the electrodeposition, and 2 M (mol L^−1^) ZnSO_4_ aqueous solution was used as the electrolyte. The electrodeposition was conducted with a constant current density of −40 mA cm^−2^ for 5 min at room temperature (corresponding to an areal capacity of 3.33 mAh cm^−2^).

#### 2.1.3 Synthesis of VOH and VOH Cathode

Vanadium-based oxide V_10_O_24_·12H_2_O (VOH) was synthesized via hydrothermal method according to previous report (Liu W. et al., 2019). Typically, 1 g V_2_O_5_ and 1 g sucrose were mixed in 70 ml deionized water. Afterward, the mixture was stirred for 1 h and transferred to a Teflon-lined autoclave. Then the autoclave was heated at 100°C for 12 h. The VOH sample was collected by filtration, washed with deionized water, and dried at 60°C for 12 h. To prepare the VOH cathode, the synthesized VOH powder was mixed with acetylene black and PVDF with a weight ratio of 7: 2: 1 in NMP solvent, then coated onto a stainless-steel current collector and dried at 80°C overnight. The mass loading of VOH cathode is about 1.4 mg cm^−2^, corresponding to an NP ratio (mass) of 2.86.

#### 2.1.4 Battery Assembly

CR2032 coin-type cells were assembled for electrochemical tests. For the galvanostatic cycling test, symmetric cells were assembled by using Zn@CC and Zn@PTCC anodes, air-laid paper separator, and 2 M ZnSO_4_ aqueous electrolyte. For the zinc plating/stripping test, zinc foil was used as counter and reference electrodes, and CC or PTCC was employed as working electrodes. The stripping cutoff voltage was set at 0.5 V (vs. Zn^2+^/Zn) for each cycle. For Zn|VOH full cell, VOH cathode was employed as the cathode, Zn@CC and Zn@PTCC were used as the anodes, and 2 M Zn(CF_3_SO_3_)_2_ was used as electrolyte.

### 2.2 Characterizations

X-ray diffraction (XRD) was performed by Bruker D8 Advance diffractometer (Cu Kα radiation, λ = 1.54056 Å) at a scan rate of 5° min^−1^. The morphology and microstructure measurements were carried out with a field emission scanning electron microscope (FE-SEM, HITACHI S4800) with energy-dispersive X-ray spectroscopy (EDS). X-ray photoelectron spectroscopy (XPS) analysis was performed through PHI 5000 VersaProbe II with Al Ka radiation. FTIR tests were conducted with Thermo Fisher Scientific FTIR spectrophotometer (Nicolet iS50). Nitrogen adsorption/desorption isotherms were obtained at 77 K by an automated adsorption apparatus (Micromeritics ASAP 2020). The surface area and pore size distribution were calculated based on the Brunauer–Emmett–Teller (BET) equation and density functional theory methods. Contact angle measurements were conducted using a drop shape analyzer (KRUSS DSA30S). Cyclic voltammetry (CV) and electrochemical impedance spectroscopy (EIS) tests were carried out on a VMP3 multichannel electrochemical station (Bio-Logic Science Instruments SA). Galvanostatic charge-discharge (GCD) tests were performed on a LAND CT 2001A battery test system.

## 3 Result and Discussion

### 3.1 Characterizations of CC and Plasma Treated Carbon Cloth

**FIGURE 1:**
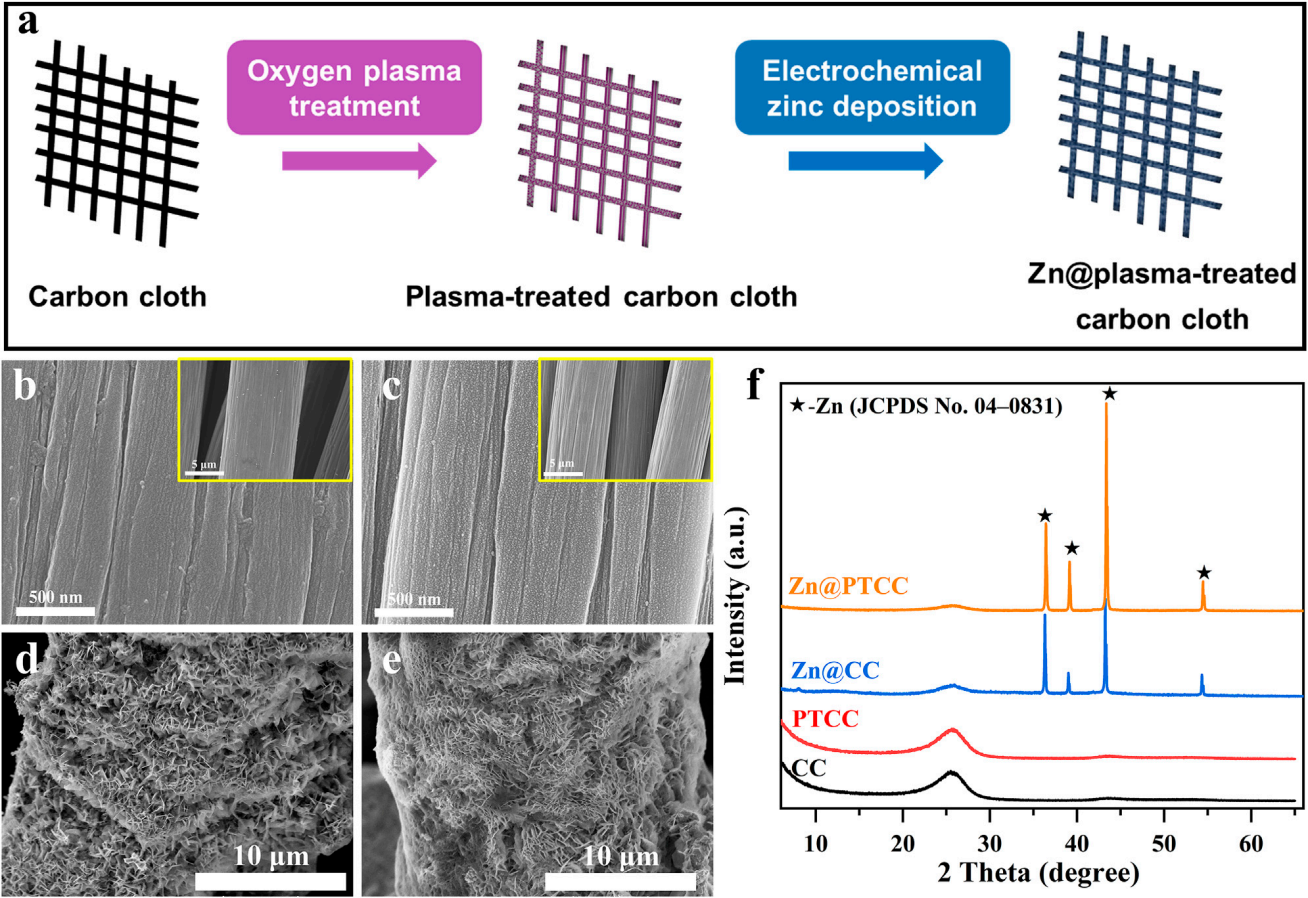
**(A)** Illustration of the synthesis process of PTCC sample and Zn@PTCC electrode. SEM images of pristine CC **(B)**, PTCC **(C)**, Zn@CC **(D)** and Zn@PTCC **(E)**. **(F)** XRD patterns of CC, PTCC, Zn@CC and Zn@PTCC.

The synthesis process of the PTCC substrate and Zn@PTCC electrode is shown in Figure 1A. The pristine carbon cloth sample was treated by oxygen plasma to obtain the PTCC sample. The surface morphology of CC and PTCC samples was characterized by scanning electron microscope (SEM). The SEM image of CC sample (Figure 1B) indicates that carbon cloth is made up of bundles of woven carbon fibers and the diameter of each carbon fiber is about 10 μm. The carbon fiber of CC sample has clean and relatively smooth surface morphology. As a comparison, numerous uniformly distributed tiny pits were observed on the carbon fiber surface of PTCC sample (Figure 1C). The appearance of these pits is related to the etching effect of carbon material induced by oxygen plasma treatment (Song et al., 2011; Chen et al., 2020). Nitrogen sorption isotherms of CC and PTCC samples (Supplymentary Figure S1) were measured to acquire the specific surface area information. The specific surface areas of CC and PTCC samples are calculated to be 0.3094 and 0.3138 m^2^ g^−1^, respectively. The results of the specific surface area tests indicate that the plasma treatment hardly changed the specific surface area of CC and PTCC samples. Zinc was then electrodeposited on CC and PTCC samples to form Zn@CC and Zn@PTCC electrodes (See Experimental Section). SEM images of Zn@CC and Zn@PTCC are shown in Figures 1D, E, respectively. After zinc deposition, zinc nanosheets with 10–100 nm in thickness were grown on the surface of CC and PTCC samples in a relatively uniform manner. The structure formed by stacking the zinc nanosheets can ensure a low-resistance path for electron transmission (Guo et al., 2020). The phase structure and phase evolution were measured by X-ray diffraction (XRD). Figure 1F shows the XRD spectroscopies of CC, PTCC, Zn@CC, and Zn@PTCC samples. Both CC and PTCC samples show the same peak at 2*θ* of 26.2°, which corresponds to the characteristic peak of amorphous carbon. The PTCC sample displays a similar XRD pattern with the CC sample, indicating that oxygen plasma treatment did not change the phase of the CC sample. The XRD patterns of the Zn@CC and Zn@PTCC samples after zinc deposition show clear diffraction peaks at 2*θ* of 36.3°, 39.0°, 43.2°, and 54.3°, which match well with metallic hexagonal zinc (JCPDS 04-0831), indicating that metallic hexagonal zinc was successfully deposited on CC and PTCC samples without other impurities.

**FIGURE 2:**
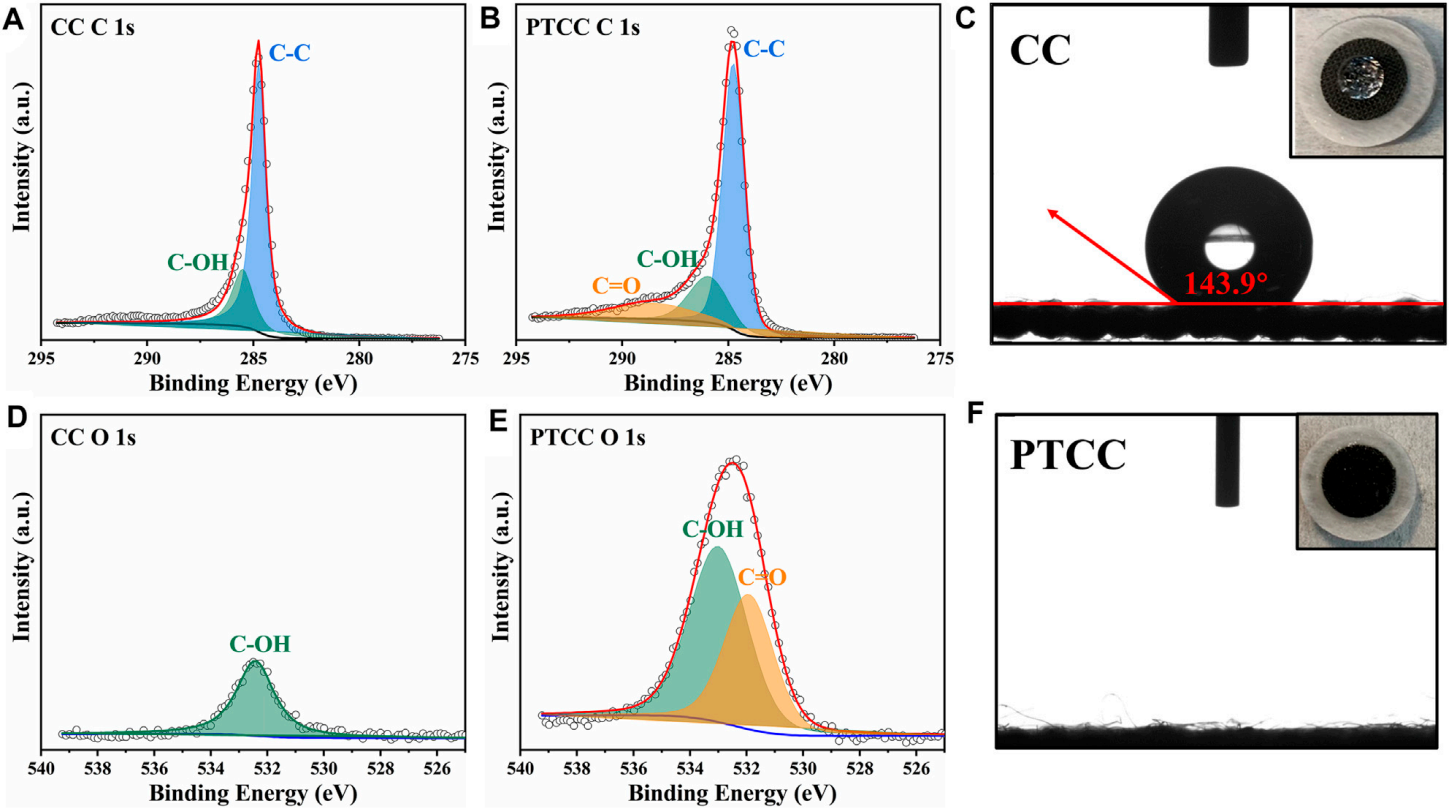
High-resolution XPS profiles of CC and PTCC samples in C 1s region **(A,B)** and O 1s region **(D,E)**. Contact angle tests of CC **(C)** and PTCC **(F)** samples.

X-ray photoelectron spectroscopy (XPS) characterization was performed to study the evolution in the surface chemical state of the samples. The XPS survey spectrums of CC and PTCC samples are shown in Supplymentary Figure S2. The survey scan proves the presence of carbon and oxygen core peaks in both CC and PTCC samples. The content of O element in the CC sample surface is 3.41%, which increases to 13.03% in the PTCC sample, demonstrating that oxygen plasma treatment has an oxidizing effect on CC samples. The de-convoluted high-resolution multiplex C 1s scan of CC and PTCC samples are displayed in Figures 2A,B. Compared to PTCC sample, only peaks of C-OH bond (285.5 eV) and C-C bond (284.7 eV) were observed on the C 1s spectrum of CC sample. The C 1s spectrum of the PTCC sample showed a novel peak at 288.5 eV, indicating that a novel chemical bond of C=O bond (carbonyl group) was created after plasma treatment. On the O 1s spectrum of CC sample (Figure 2D), just one peak of C-OH was observed at 532.3 eV, but a new peak of C=O appeared at 531.9 eV on the O 1s spectrum of PTCC sample (Figure 2E). The XPS analysis result reveals that carbonyl groups were effectively introduced to the surface of PTCC sample by oxygen plasma treatment, which is also verified by the corresponding FTIR analysis (Supplymentary Figure S3). The FTIR spectrum displays a peak around 1720 cm^−1^, which corresponds to the C=O stretching bonds. In addition, the distribution of carbonyl groups in the PTCC sample was also analyzed by energy-dispersive X-ray spectroscopy. The EDS mapping images of PTCC sample (Supplymentary Figure S4) exhibit a homogeneous dispersion of oxygen element, indicating that the C=O functional groups were uniformly distributed on the PTCC surface. The abundant carbonyl groups on the surface of PTCC electrode can enhance the hydrophilicity of the sample surface and help to improve the wettability of the electrolyte to the electrode (Cho et al., 2000; Cha et al., 2019). Contact angle tests were performed to evaluate the electrolyte wettability of CC and PTCC electrodes. As shown in Figures 2C, F, PTCC sample exhibits significantly improved wettability of aqueous electrolyte (2 M ZnSO_4_ aqueous solution) than CC sample. The contact angle of CC sample is measured to be 143.9°, which corresponds to a hydrophobic surface. For PTCC sample, the electrolyte droplet penetrates instantly into the PTCC after the droplet reaches its surface, which represents a near-zero contact angle. The optimized wettability of the electrolyte demonstrates a decrease in the interfacial free energy between the electrode surface and the electrolyte, which facilitates the uniform distribution of electrolyte flux towards the electrode (Liu M. et al., 2019). At the same time, owing to the high binding energy between C=O and Zn, the carbonyl groups can also act as nucleation sites to guide the uniform zinc deposition (Zhou et al., 2022).

### 3.2 Electrochemical Performance Evaluation

**FIGURE 3:**
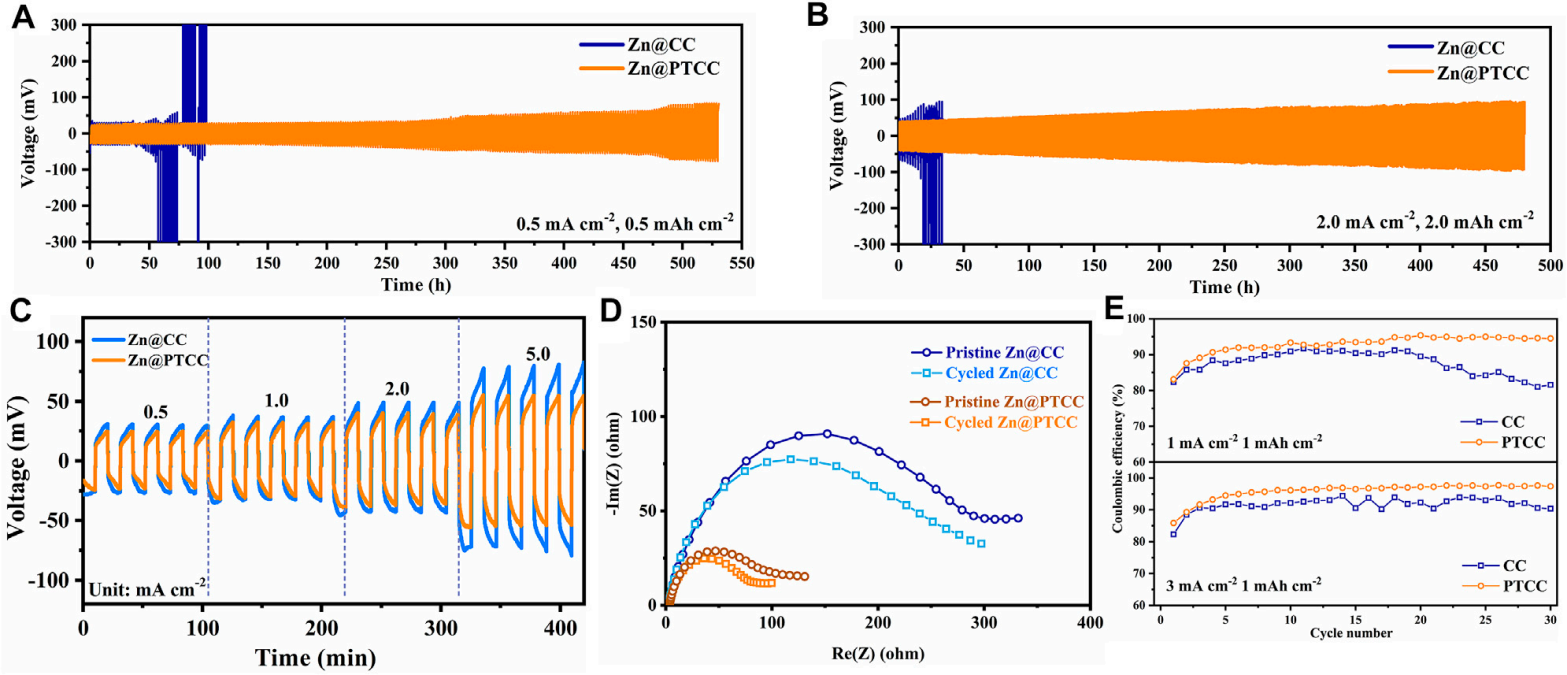
Cycling performances of the symmetrical cells with Zn@CC and Zn@PTCC electrodes at 0.5 mA cm^−2^ **(A)** and 2.0 mA cm^−2^ **(B)**. **(C)** Rate performance of the symmetric cells at current densities from 0.5 to 5.0 mA cm^−2^. **(D)** EIS profiles of the symmetric cells in pristine state and cycled state. **(E)** Coulombic efficiencies of the zinc plating/stripping on CC and PTCC electrodes at different current densities.

The electrochemical performance of Zn@CC and Zn@PTCC anodes was investigated in symmetrical cells. To determine the long-term cycling stability, Zn@CC and Zn@PTCC symmetrical cells were cycled at various current densities with 2 M ZnSO_4_ electrolyte. As shown in Figure 3A, the Zn@PTCC electrode presents stable voltage profile with a low voltage hysteresis of about 25 mV and more than 530 h lifespan at a current density of 0.5 mA cm^−2^ with a limited capacity of 0.5 mAh cm^−2^ (∼15% DOD). In contrast, the voltage profile of the Zn@CC electrode shows significant voltage fluctuation after 35 h of cycling, and then the cell fails, which is related to the short circuit caused by zinc dendrite growth (Li et al., 2022). The remarkable durability of Zn@PTCC anode can be validated by cycling at a higher current density of 2 mA cm^−2^ and higher capacity of 2 mAh cm^−2^ (Figure 3B). The Zn@PTCC anode enables the symmetric cell to be stably cycled at a lower voltage hysteresis (∼40 mV) without short circuits over 480 h with a very high DOD (∼60%). The rate performance of Zn@CC and Zn@PTCC electrodes at a variety of current densities is shown in Figure 3C. At various current densities, particularly at high current densities, the Zn@PTCC electrode exhibits significantly less voltage hysteresis than the Zn@CC electrode, indicating reduced polarization and enhanced stability. Electrochemical impedance spectroscopy (EIS) were used to further characterize the interfacial charge transfer resistance of symmetric cells. The EIS tests of Zn@CC and Zn@PTCC cells were recorded in the frequency range from 10 mHz to 300 kHz with an amplitude of 5 mV (Figure 3D). The Zn@PTCC symmetrical cell exhibits considerably lower charge transfer resistance than that of Zn@CC symmetrical cell in both pristine state and cycled state (after 10 h cycling), indicating the superior charge transfer kinetics in Zn@PTCC electrode. Asymmetric Zn|CC and Zn|PTCC cells were assembled to explore the zinc plating/stripping behaviors on CC and PTCC electrodes. As shown in Figure 3E, at a current density of 1 mA cm^−2^, the Coulombic efficiency of the CC electrode first increases, then decreases, and decays to 81.57% after 30 cycles. As a comparison, the CE of the PTCC electrode can be maintained at about 93–95%. When the current density increases to 3 mA cm−2, the PTCC electrode exhibits an average CE of about 97.42%, exceeding that of the CC electrode (92.21%). The voltage profiles of zinc plating/stripping on CC and PTCC electrodes at a current density of 3 mA cm−2 are presented in Supplymentary Figure S5. The discharge curves of PTCC electrode exhibits higher capacities than that of CC electrode, which is in accordance with the higher CE. The stable and high CE of the PTCC electrode demonstrates enhanced reversibility of zinc plating/stripping on the surface of the PTCC electrode.

### 3.3 Mechanism Investigation

**FIGURE 4:**
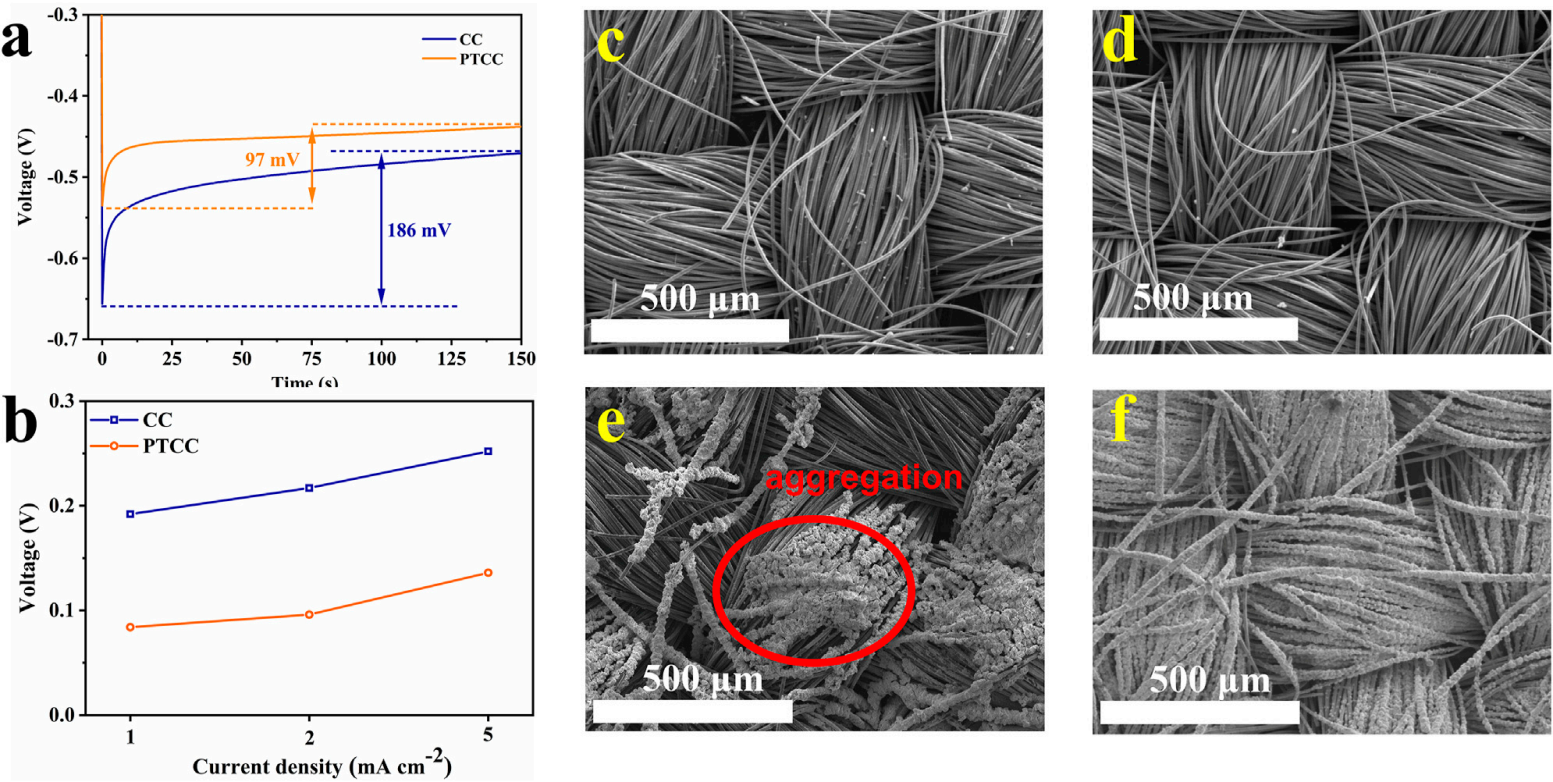
**(A)** The voltage-time curves of zinc nucleation on CC and PTCC electrodes at a current density of 1 mA cm^−2^. **(B)** Nucleation overpotentials of zinc on CC and PTCC electrodes at different current densities. SEM images of CC and PTCC electrodes before **(C–D)** and after **(E–F)** zinc deposition.

The deposition of zinc metal on the substrate usually consists of two stages: nucleation and deposition. The nucleation overpotentials of CC and CNT electrodes were compared to investigate the mechanism of PTCC electrodes in regulating zinc nucleation behavior. The PTCC electrode exhibits a reduced nucleation overpotential (97 mV) compared to the CC electrode (186 mV) at a current density of 1 mA cm^−2^ (Figure 4A). Even at high current densities (2 and 5 mA cm^−2^), the PTCC electrodes still demonstrate lower nucleation overpotential (Figure 4B). The reduction in the nucleation overpotential manifests that the PTCC electrode has lower resistance of zinc nucleation, which is related to the improved electrolyte wettability and enhanced zincophilicity of the electrode. According to density functional theory (DFT) calculations (Supplymentary Figure S6), C=O bond (−0.38 eV) has higher binding energy to Zn atom than that of C-C (−0.20 eV) and C-OH (−0.25 eV) bonds, indicating that C=O has a good ability to capture Zn ions, thereby lowering the Zn ion transport barrier (Li et al., 2020). The uniformly distributed C=O functional groups on the surface of PTCC sample not only improve the wettability of the aqueous electrolyte but also acts as zincophilic active sites to promote uniform zinc nucleation and deposition, which leads to the improvement in the electrochemical performance of the PTCC electrode.

To demonstrate the significant differences in electrochemical properties between CC and PTCC electrodes, the zinc plating morphology on CC and PTCC electrodes after zinc deposition was characterized by SEM to evaluate their resistance to zinc dendrite growth. The SEM images of CC and PTCC electrodes before zinc deposition are shown in Figures 4C, D. It can be seen that CC and PTCC have similar fibrous textile morphology. After zinc deposition, Zn@CC and Zn@PTCC samples exhibit quite different morphologies. Zinc metal is uniformly deposited on the surface of each carbon fiber (Figure 4F). On the contrary, zinc aggregations in a small area of the electrode were observed on the surface of Zn@CC sample (Figure 4E). The aggregation is related to inhomogeneous zinc nucleation and deposition, which would evolve into zinc dendrite growth and lead to further safety problems. The substantial difference suggests that PTCC electrodes are more resistant to zinc dendrite growth, which correlates with their reduced nucleation overpotential and uniformly distributed nucleation sites.

**FIGURE 5:**
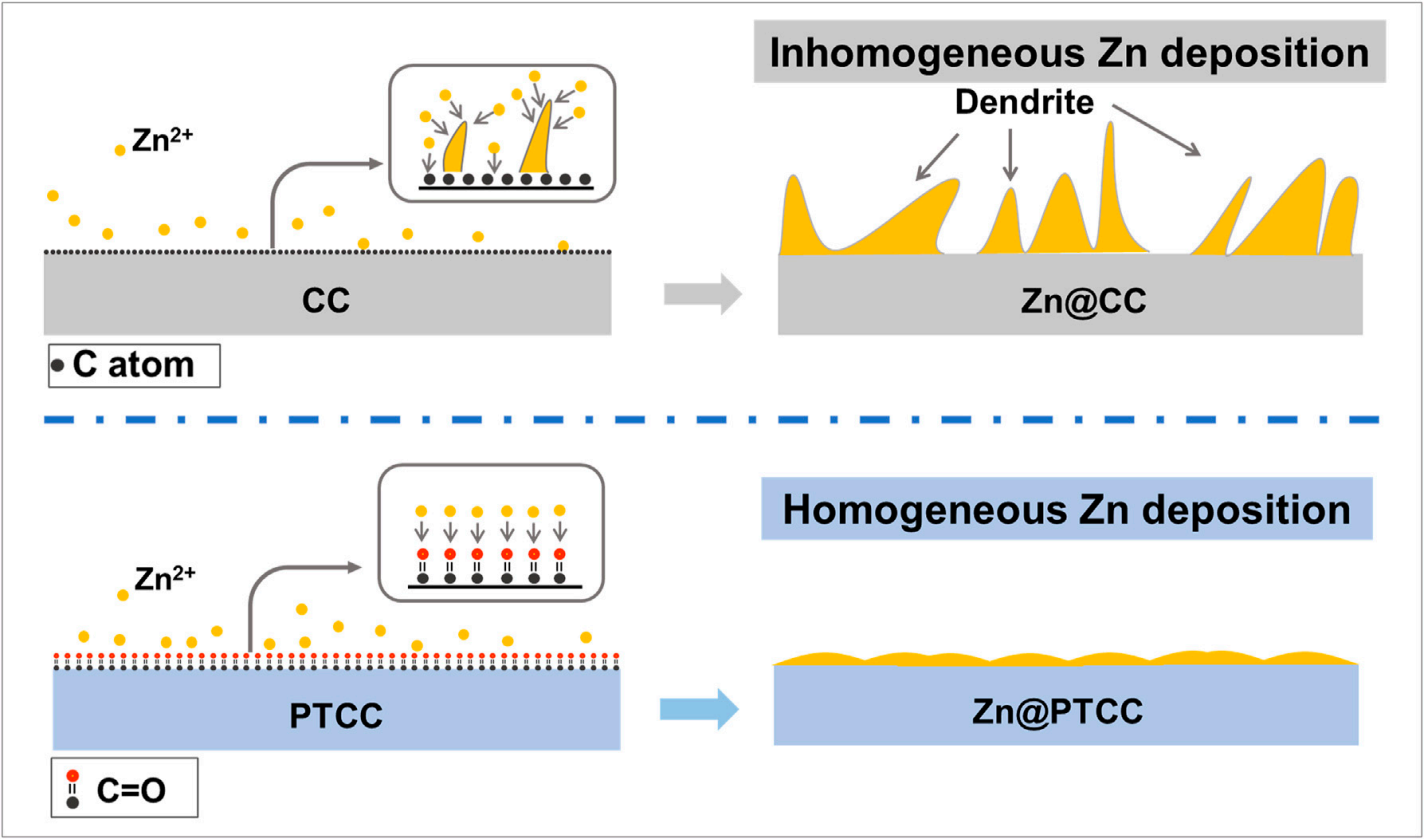
Schematic illustration of zinc plating behaviors on CC and PTCC electrodes.

The schematic comparison of zinc plating behaviors on the CC and PTCC electrodes is shown in Figure 5. Compared to the CC electrode, the PTCC electrode exhibit enhanced electrochemical performance and uniform zinc deposition without dendrite growth. Due to the improper electrolyte wettability and poor zincophilicity of the CC electrode, at the early nucleation stage, zinc cannot be nucleated uniformly on the electrode surface. As a result, zinc is nucleated and deposited in a small area. With further zinc deposition, zinc protrusions and aggregation will generate on the CC electrode surface. Once the zinc protrusions appear on the CC surface, the concentrated electric field will further lead to increased zinc dendrite growth and dead zinc (Xie et al., 2021). As a comparison, the PTCC electrode has better electrolyte wettability, enhanced zinc affinity and uniformly distributed zincophilic sites for zinc nucleation. Therefore, zinc would uniformly nucleate onto the PTCC surface at the early stage, and no dendrite growth was triggered during the subsequent deposition process.

### 3.3 Full-Battery Evaluation

**FIGURE 6:**
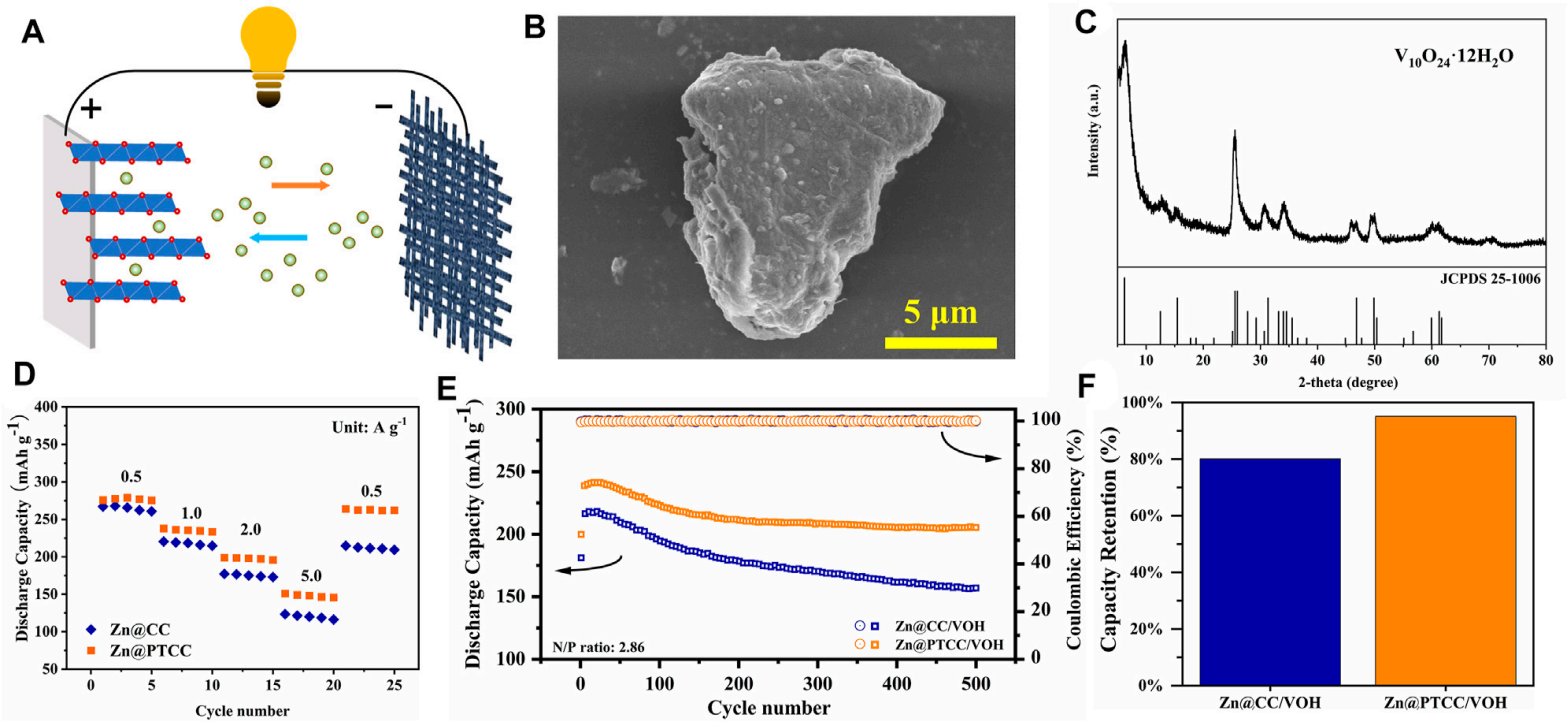
**(A)** Schematic illustration of Zn|VOH full battery. SEM image **(B)** and XRD pattern **(C)** of the VOH material. Rate performance **(D)** and cycling performance at a current density of 1 A g^−1^ **(E)** of the Zn|VOH batteries with Zn@CC and Zn@PTCC anodes. **(F)** Capacity retention comparison for the Zn|VOH batteries.

As displayed in Figure 6A, full cells with vanadium-based oxide V_10_O_24_·12H_2_O (VOH) as cathode were assembled to evaluate the practicability of the Zn@PTCC electrode. The VOH cathode material was synthesized via hydrothermal method. The morphology of the VOH material is depicted in Figure 6B, displaying that it is composed of micron-sized particles. The XRD diffraction analysis (Figure 6C) confirms the sample as V_10_O_24_·12H_2_O (JCPDS No.25-1006) with extra-large layer spacing of 1.42 nm, stabilized by interlayer water molecules. Then, rate performance and long-term cycling stability tests were performed in full cells. The rate capabilities of Zn|VOH full cells with Zn@CC and Zn@PTCC anodes at different current densities are shown in Figure 6D. The rate capability was improved in the full cell with Zn@PTCC anode. Compared to the full cell with Zn@CC anode, the Zn@PTCC full cell exhibits higher capacity at all current densities. The long-term stability of Zn@CC|VOH and Zn@PTCC|VOH full cells at a current density of 1 A g^−1^ is presented in Figure 6E. Both Zn@CC and Zn@PTCC cells exhibit a gradual increase in capacity during certain initial cycles. This phenomenon could be attributed to the activation process of vanadium-based oxides at the initial stage (Liu W. et al., 2019). The Zn@PTCC|VOH cell exhibits a reversible capacity of 216.0 mAh g^−1^, which increases to the maximum value of 318.8 mAh g^−1^ at the 25th cycle and remains 205.3 mAh g^−1^ after 500 cycles, corresponding to a capacity retention of 95.0%. In contrast, the capacity retention of Zn@CC|VOH cell after 500 cycles is only 80.1% (Figure 6F). The improvement in reversible capacity and capacity retention confirms the enhanced cycling durability of the Zn@PTCC anode.

## 4 Conclusion

In summary, oxygen plasma was used to modify the interfacial chemical state of carbon cloth to develop an ideal substrate for zinc deposition to solve the dendrite growth problem of zinc anodes. The plasma treatment introduced uniformly distributed C=O functional groups onto the carbon cloth surface, improving the electrolyte wettability and zincophilicity of the substrate, resulting in a decreased nucleation overpotential. Meanwhile, the uniformly distributed C=O functional groups can also act as active sites for zinc nucleation to guide the uniform nucleation and deposition of zinc. The Zn@PTCC anodes demonstrated low voltage hysteresis (25 mV) and stable zinc plating/stripping behaviors (over 530 h lifespan with ∼15% DOD) at a current density of 0.5 mA cm^−2^. In addition, the symmetric cell could maintain a stable cycle life (480 h) even at very high DOD of 60%. When paired with V_10_O_24_·12H_2_O cathode, the full cell with Zn@PTCC anode exhibited superior cycling stability (95.0% capacity retention after 500 cycles), much higher than that with Zn@CC anode (80.1%). Compared with other common artificial interface construction strategies, this plasma treatment method is more facile and versatile. The species and amount of the functional groups to be introduced can also be precisely controlled. Besides, this plasma treatment method can also be applied to other carbon-based or organic substrates, which can provide new insights into the design and modification of stable substrates for rechargeable metal anodes.

## Data Availability

The original contributions presented in the study are included in the article/Supplementary Material, further inquiries can be directed to the corresponding authors.
