# Supplementary material for: Oxygen Plasma Modified Carbon Cloth with C=O Zincophilic Sites as a Stable Host for Zinc Metal Anodes
Source: Front Chem. 2022 Apr 28;10:899810. doi: 10.3389/fchem.2022.899810 (PMC9096248; doi:10.3389/fchem.2022.899810)
Supplement: Supplementary file 1 [file DataSheet1.PDF]

Supporting Information

Oxygen Plasma Modified Carbon Cloth with C=O Zincophilic Sites as a Stable Host for Zinc Metal Anodes

Baozheng Jiang[a], Wenbao Liu[b], Zhilong Ren[a], Rongsheng Guo[a], Yongfeng Huang[a], Chengjun Xu[a,*], Feiyu Kang[a,c,*]

a Shenzhen Geim Graphene Center, Tsinghua-Berkeley Shenzhen Institute & Tsinghua Shenzhen International Graduate School, Tsinghua University, Shenzhen 518055, China

b School of Environmental and Material Engineering, Yantai University, Yantai 264005, China

c State Key Laboratory of New Ceramics and Fine Processing, School of Materials Science and Engineering, Tsinghua University, Beijing 100084, China

*** Corresponding Authors:**

zincgroup@126.com (C. Xu); fykang@mail.tsinghua.edu.cn (F. Kang)

Supporting Information

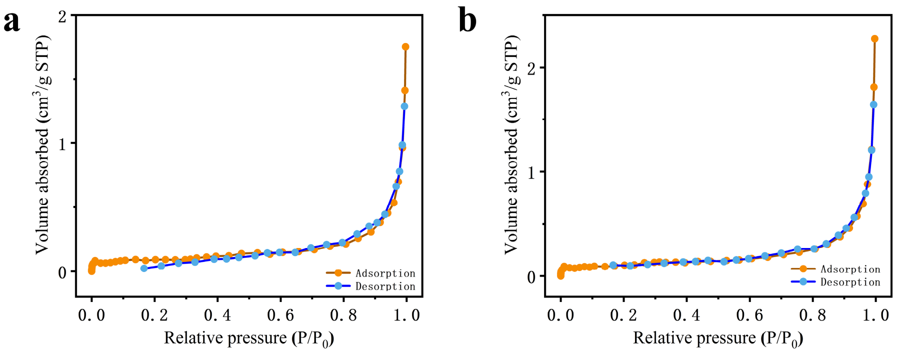

Figure S1. Nitrogen sorption isotherms of CC (a) and PTCC (b) samples.

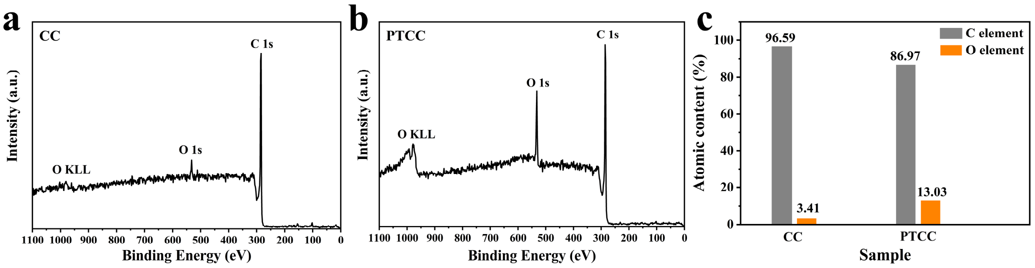

Figure S2. XPS survey scans of Zn@CC (a) and Zn@PTCC (b) samples. (c) C and O element content in the surface of CC and PTCC samples.

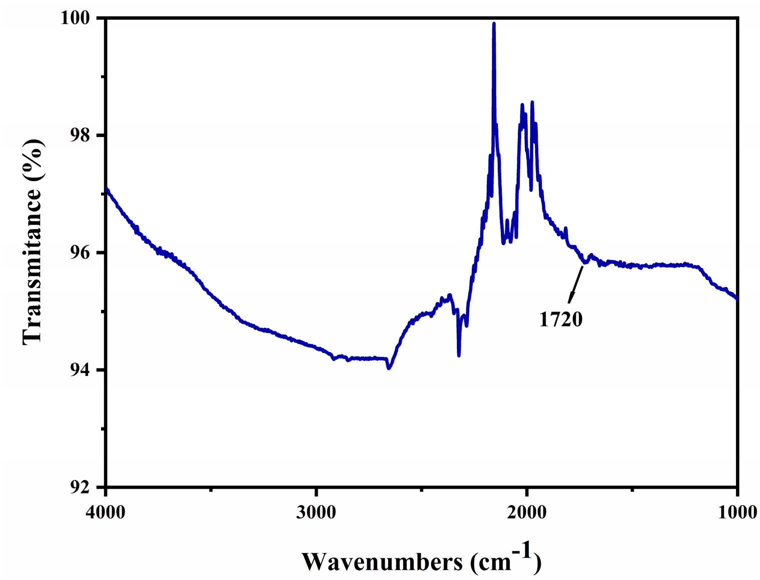

Figure S3. FTIR spectrum of the PTCC sample.

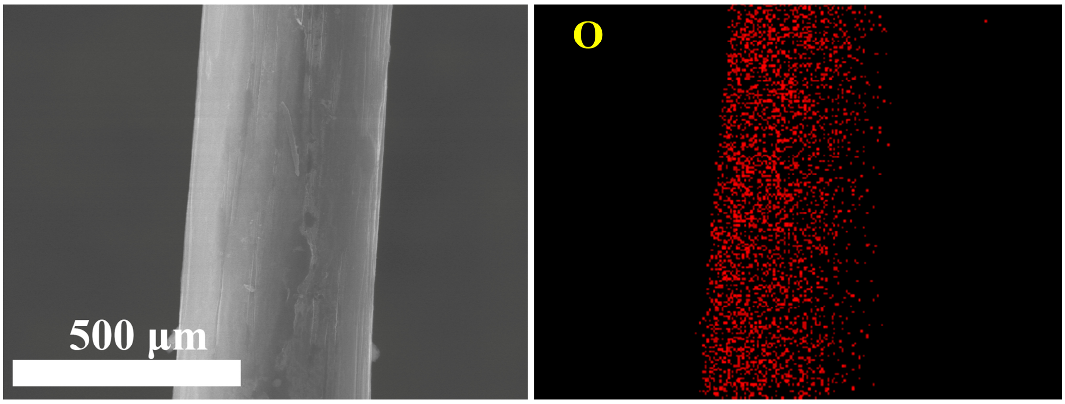

Figure S4. EDS mapping of O element in the PTCC sample.

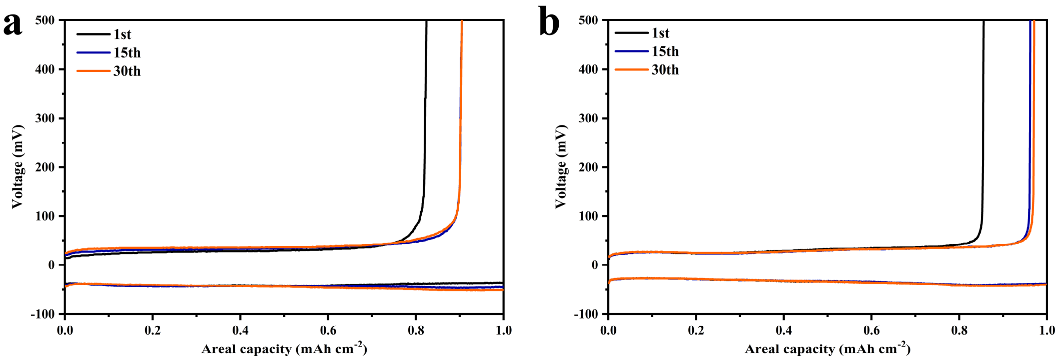

Figure S5. Voltage profiles of zinc plating/stripping on CC (a) and PTCC (b) electrodes at a current density of 3 mA cm^{-2} with a limited capacity of 1 mAh cm^{-2}.

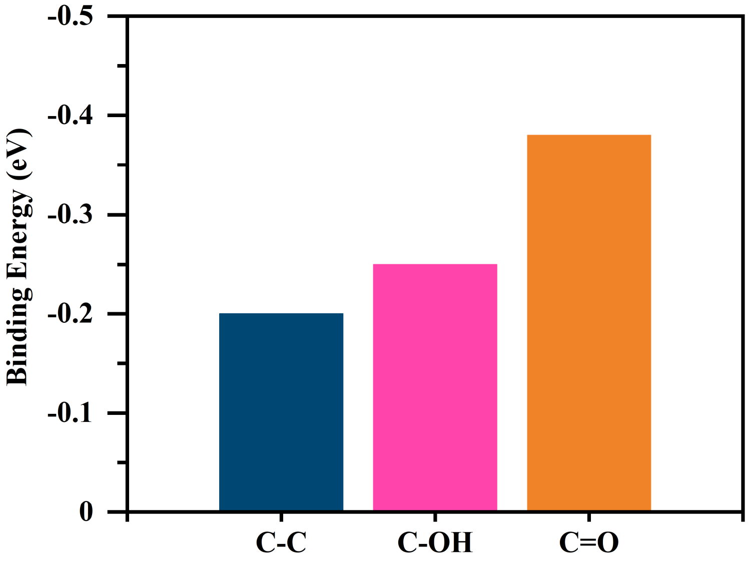

Figure S6. The binding energy of zinc atom for different functional groups on carbon material. (Li et al., 2020)

[1] Li, C., Sun, Z., Yang, T., Yu, L., Wei, N., Tian, Z., et al. (2020). Directly Grown Vertical Graphene Carpets as Janus Separators toward Stabilized Zn Metal Anodes. *Adv Mater* 32(33)**,** e2003425. doi: 10.1002/adma.202003425.
